# A large predatory reef fish species moderates feeding and activity patterns in response to seasonal and latitudinal temperature variation

**DOI:** 10.1038/s41598-017-13277-4

**Published:** 2017-10-11

**Authors:** Molly Scott, Michelle Heupel, Andrew Tobin, Morgan Pratchett

**Affiliations:** 1 ARC Centre of Excellence for Coral Reef Studies, James Cook University, Townsville, QLD 4811 Australia; 2 Australian Institute of Marine Science, PMB No 3, Townsville, Queensland 4810 Australia; 3 Centre for Sustainable Tropical Fisheries and Aquaculture and School of Earth and Environmental Sciences, James Cook University, Townsville, Queensland 4811 Australia

## Abstract

Climate-driven increases in ocean temperatures are expected to affect the metabolic requirements of marine species substantially. To mitigate the impacts of increasing temperatures in the short-term, it may be necessary for ectothermic organisms to alter their foraging behaviour and activity. Herein, we investigate seasonal variation in foraging behaviour and activity of latitudinally distinct populations of a large coral reef predator, the common coral trout, *Plectropomus leopardus*, from the Great Barrier Reef, Australia. *P. leopardus* exhibited increased foraging frequency in summer versus winter time, irrespective of latitude, however, foraging frequency substantially declined at water temperatures >30 °C. Foraging frequency also decreased with body size but there was no interaction with temperature. Activity patterns were directly correlated with water temperature; during summer, the low-latitude population of *P. leopardus* spent up to 62% of their time inactive, compared with 43% for the high-latitude population. The impact of water temperature on activity patterns was greatest for larger individuals. These results show that *P. leopardus* moderate their foraging behaviour and activity according to changes in ambient temperatures. It seems likely that increasing ocean temperatures may impose significant constraints on the capacity of large-bodied fishes to obtain sufficient prey resources while simultaneously conserving energy.

## Introduction

Sustained and ongoing ocean warming^1^, is exposing marine organisms to unprecedented and ever-increasing temperatures. For ectothermic animals, such as fishes, temperature fundamentally affects individual metabolic rates, which influence growth, reproduction, movement, behaviour, and consequently fitness and survival^2–4^. Metabolic performance and function are underpinned by the uptake, transport, and delivery of oxygen throughout an organism’s tissue^5,6^. For fishes, metabolic capacity is ultimately constrained by oxygen delivery^7^ and at high temperatures, this limitation is often compounded by declines in oxygen availability and increases in oxygen demand^8^. Temperature-driven changes in oxygen budgets can compromise the respiratory energy available for fitness and performance^9^ and at higher temperatures individuals may be forced to adopt energy-saving strategies, which may lead to reductions in energetically demanding activities, such as swimming and foraging^10,11^.

The vulnerability of populations and species to changing environmental regimes will be determined by their ability to adapt^12^, acclimate or acclimatise^13^. Adaptation is genetic change that occurs across generations or among populations in relation to environmental change^14^. Acclimation refers to short-term changes in behaviour, physiology, or both, that arise in an individual in response to a single environmental variable^15^. Acclimatisation is a behavioural or physiological response to multiple environmental variables, typically recorded under field conditions^15^. Short-term temperature fluctuations can directly influence an organism’s capacity for acclimation or acclimatisation through the impact on physiological reaction rates. Individuals may alter behavioural patterns and fitness if they cannot compensate physiologically for temperature variability^14^. For example, it has been shown that fishes change their foraging behaviour and increase food intake and time spent feeding to compensate for increases in metabolic demands at higher temperatures^11,16,17^. Fishes may also increase their time spent resting to regulate increasing metabolic costs at higher temperatures^10,18^. Crucially, any modifications to behaviour may come at a cost to the individual and consequently, the ecosystem. For example, movement and activity are directly related to prey encounter and predator evasion^19^, and alterations may therefore limit food intake and increase susceptibility to predation^20^. Alternatively, an increase in food intake may cause potential distortion to food webs, as intake may not be met by greater production at lower trophic levels^11^. Changes in behavioural patterns, therefore, may not only affect individual fitness, but also species interactions, population dynamics, community structure and ultimately biodiversity and ecosystem function^21,22^.

Tropical species are considered to be more vulnerable to increasing temperatures than temperate counterparts because they generally experience limited diurnal and seasonal variation in temperature^23,24^. In shallow coral reef ecosystems, for example, seasonal ocean temperatures may only differ by up to 5–6 °C annually^25^, although localised variations may be greater, particularly for shallow, lagoonal waters. In contrast, temperate waters regularly vary up to 10 °C annually^25^. This means that for coral reef species, small increases in ambient water temperature may subject individuals and populations to unprecedented temperatures, leading to a greater energetic cost of maintaining standard metabolic activity^26,27^. Experimental studies conducted on a variety of coral reef fishes demonstrate that some species are already living close to their thermal optima^28,29^ and are likely to be negatively impacted by projected increases in ocean temperatures^30^. Ultimately, however, species response to ocean warming depends on how populations are affected by increasing temperature throughout their geographic range.

Studies of latitudinally distinct populations of fishes have revealed differences in thermal tolerances suggestive of local acclimation^12,29^. Notably, low-latitude populations subjected to higher summertime temperatures can tolerate higher temperatures and often perform best at higher temperatures, compared to conspecifics from higher latitudes^12,29^. For common coral trout (*Plectropomus leopardus*), however, there does not appear to be any difference in thermal sensitivity (i.e. sensitivity to variations in water temperatures), between fish sampled from latitudinally distinct locations where average maximum summer temperatures differ by up to 3 °C^31^. Individuals from both populations exhibited declines in performance when subjected to water temperatures >30 °C^10,32^. These findings suggest that populations of *P. leopardus* on the Great Barrier Reef (GBR) are poorly acclimated to local temperature regimes, possibly due to high levels of genetic exchange at the scale of the entire GBR^32^. As such, sustained increases in ocean temperatures may already compromise body condition and physiological performance of *P. leopardus* at low latitudes (where summertime temperatures already exceed 30 °C)^33^. Conversely, larger, more mobile fishes, such as *P. leopardus* may have greater capacity to mediate exposure to high summertime temperatures by exploiting natural gradients (e.g., with depth) in environmental conditions, thereby concealing any capacity for local acclimation. *P. leopardus* may also compensate for temperature-induced increases in metabolism by modifying their food intake^11^, provided food is not limited by prey availability or abundance^34^, and through a reduction in swimming activity^10^.

Experimental studies are likely to overestimate the impacts of higher temperatures on wild populations of fishes because they generally expose individuals to rapid and pronounced changes in temperature, undermining any capacity for acclimation^3,10,29^ and also fail to account for the ability of fishes to behaviourally mediate exposure to increasing temperatures^35,36^. *In situ* behavioural plasticity may also be limited by the abiotic environment, such as water quality and topography. Consequently, there is a need to understand whether large-bodied, coral reef predators can mediate exposure to environmental changes through modification of their behaviour. Coral trout, *Plectropomus* spp., are commercially and economically important fisheries species on the GBR. They are relatively mobile with predicted home ranges of 0.5 km^2^ ^37^, and are an ecologically important mesopredator, with a primarily generalist, piscivorous diet^38^.

This study explores seasonal variation in the *in situ* foraging behaviour and activity of *P*. *leopardus* at two latitudinally distinct locations on Australia’s GBR separated by approximately 1,200 km. Specifically, this study quantifies spatial and temporal differences in strike rates (and predation success) as well as activity patterns (i.e. the time spent resting or inactive) for *P. leopardus*. We expected that *P. leopardus* would exhibit seasonal and latitudinal differences in foraging behaviour and activity corresponding to differences in water temperature. Specifically, fish were expected to respond to moderate increases in temperature by increasing foraging activity and food intake. Increases in foraging activity would, however, require greater energy investment and movement, further increasing metabolic demands. Therefore, it is likely that there will be a tradeoff between food intake and conservation of energy that will constrain the extent to which individuals can increase foraging activity at high temperatures. It is also expected that responses of *P. leopardus* to increasing temperature will be strongly size-dependent, whereby larger-bodied individuals are more thermally sensitive than smaller individuals^39^. We predict, therefore, that larger-bodied individuals will exhibit more pronounced seasonal and latitudinal differences in foraging behaviour and activity, though it is also likely that larger fishes feed less often^11^.

## Results

*In situ* observations of foraging behaviour and activity were undertaken for *P. leopardus* in summer (February–March 2016) and winter (July–August 2016) at Lizard Island (14°40′S, 145°27′E) in the northern GBR (low-latitude population) and Heron Island (23°29′S, 151°52E) in the southern GBR (high-latitude population). Observations were carried out between 0700–1730 hrs to test for diurnal variation in foraging and activity. Ambient temperatures varied both seasonally and latitudinally, ranging from 20 °C (during winter at Heron Island) up to 32 °C (during summer at Lizard Island) (see Methods).

### Foraging behaviour

**Figure 1:**
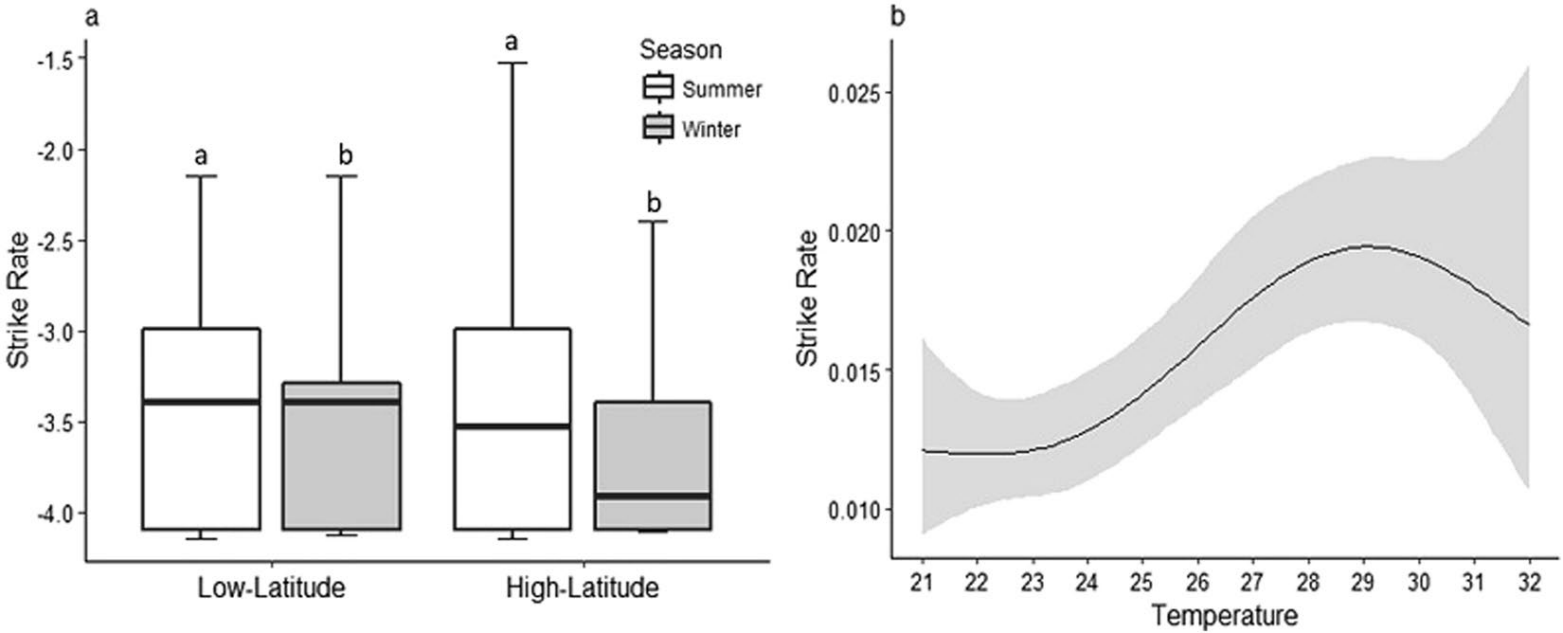
(**a**) Box plots showing log transformed average strike rates of *P. leopardus* from low-latitude and high-latitude locations (p = 0.563) during summer (white) and winter (grey) (p = 0.033). Data (n = 595) are for each individual observation. The whiskers are extended to extreme values. Boxplots with the same letter are not significantly different. (**b**) The modelled values of the relationship between strike rate and increasing temperature (p < 0.01) from a Generalised Additive Model with a smoothing function. The shaded region around the curve represents a 95% confidence interval.

A total of 486 feeding strikes were recorded across the 595 individuals observed during this study, with an average of 0.96 strikes per hour. The majority of strikes took place over coral reef habitat, compared with the water column (p = 0.003) or ‘other’ habitat (i.e. sand or algal covered rocks) (p < 0.001). Strike rates varied considerably with season, but not location (Table 1), averaging 1.14 (±0.002 SE) per hour in summer versus 0.78 (±0.001 SE) per hour in winter. Seasonal differences in strike rates were most pronounced at the high-latitude location, because of the very low winter average strike rate, 0.6 (±0.001 SE) per hour, compared with 1.2 (±0.002 SE) per hour for the low-latitude population (Fig. 1a). Whilst strike rates were highest during summer, increasing temperatures had a negative impact on strike rates of *P. leopardus*. Strike rates were highest at 30 °C and every 3 °C increase in temperature between 21 °C and 30 °C led to a 1.4 – fold increase in strike rate. Beyond 30 °C, strike rates declined, indicating the potential for a negative response of foraging activity to increases in temperature, and this effect was consistent across all body sizes (Table 2).

**Table 1:** Coefficient table of the negative binomial generalised linear model showing the influence of season, trout length, location, and time on strike rate for *P*. *leopardus* once the data have been centered for temperature. P-values (in bold) have been converted from z-scored such that significance is measured as p < 0.05.

**Strike rate**	**Estimate**	**St. Error**	**p-value**
Intercept	−2.805	0.269	**<0.001**
Season	−0.836	0.392	**0.033**
Body Size (TL)	−0.027	0.006	**<0.001**
Location	0.070	0.120	0.563
Time of day	−0.008	0.0315	0.796
Season x Body Size (TL)	0.011	0.016	0.284

**Table 2:** Generalised additive modelling; Intercept, effective degrees of freedom (edf) and significance (p-value) of temperature and body size (TL) on strike rates for *P*. *leopardus*. Coefficient of determination (R^2^), the explained deviance, and the AIC values are given for each model. Significance terms are in bold.

	**Strike Rate**				**Activity**			
	**Model 1: TEMP + BODY SIZE**		**Model 2: TEMP** * **BODY SIZE**		**Model 1: TEMP + BODY SIZE**		**Model 2: TEMP** * **BODY SIZE**	
	Estimate	p-value	Estimate	p-value	Estimate	p-value	Estimate	p-value
Intercept	−4.18	<0.001	−4.17	<0.001	0.47	<0.001	0.47	<0.001
SE	0.06		0.06		0.01		0.01	
	***edf***	**p-value**	***edf***	**p-value**	***edf***	**p-value**	***edf***	**p-value**
Temp	2.68	**0.0065**	2.44	**0.01**	6.89	**<0.001**	7.08	**<0.001**
Body Size	1.69	**<0.001**	1.80	**<0.001**	3.32	**<0.001**	1.00	**<0.001**
Temp × Body Size	—	—	1.00	0.176		—	4.73	**0.005**
R^2^	0.07		0.07		0.14		0.15	
Deviance explained	8.69%		8.85%		15.5%		17.2%	
n	595		595		595		595	
AIC	1451.4		1452.2		20.54		14.64	

**Figure 2:**
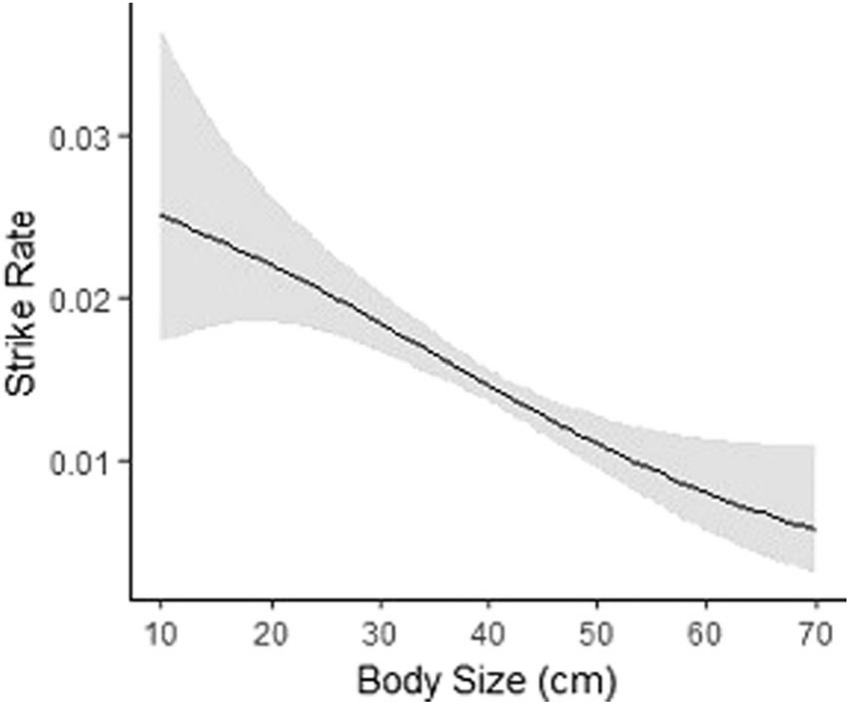
The modelled values of a Generalised Additive Model with a smoothing function demonstrating the negative relationship between strike rate and body size of *P. leopardus* (measured in total length to the nearest 5 cm). The shaded region around the curve represents a 95% confidence interval, (p < 0.001).

Although smaller individuals displayed substantially higher strike rates than larger individuals (Fig. 2), it appears that the foraging frequency of *P. leopardus* is equally compromised by higher temperature regardless of body size (Table 1).

**Figure 3:**
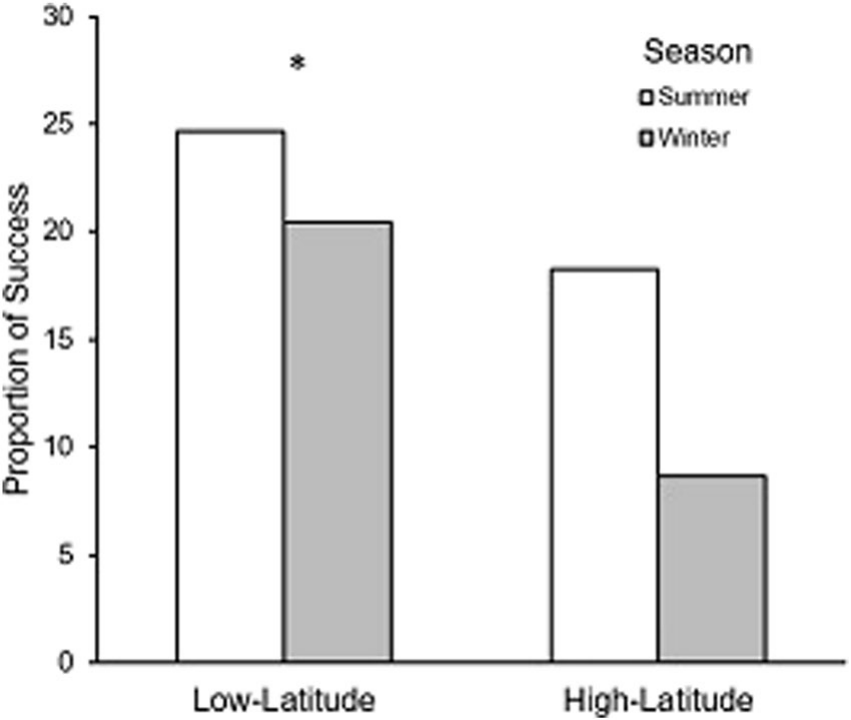
Proportion of successful strikes made by individual *P. leopardus* between low-latitude and high-latitude locations (p = 0.02) in summer (white) and winter (grey) (p = 0.87). Significant differences are marked with an *, based on a chi-squared test of proportional data.

Similarly, the proportion of successful strikes of *P. leopardus* (recorded by one observer to reduce observer bias), did not vary with body size, suggesting all size classes were equally likely to make a successful strike. Further, success rates did not differ between seasons (p = 0.87), but were significantly different between locations (p = 0.02), a pattern driven by consistently higher success rates in summer and winter of the low-latitude population (Fig. 3). Overall, of the 278 strikes made by individual *P. leopardus*, 47 were considered successful, giving an overall strike success rate of 17%. In general, the low-latitude population had a higher proportion of successful strikes than the high-latitude population. For the low-latitude population in the summer 26% of strikes were successful, compared with 16% success in the winter. For the high-latitude population strike success was 21% in summer and 10% in winter.

Surprisingly, there was no significant diurnal variation in feeding behaviour of *P. leopardus* (Fig. S 1), however, the majority of strikes were observed in the morning (0700–1100) and fewest strikes at midday at both locations (1100–1400) (Table 1).

### Activity patterns

**Figure 4:**
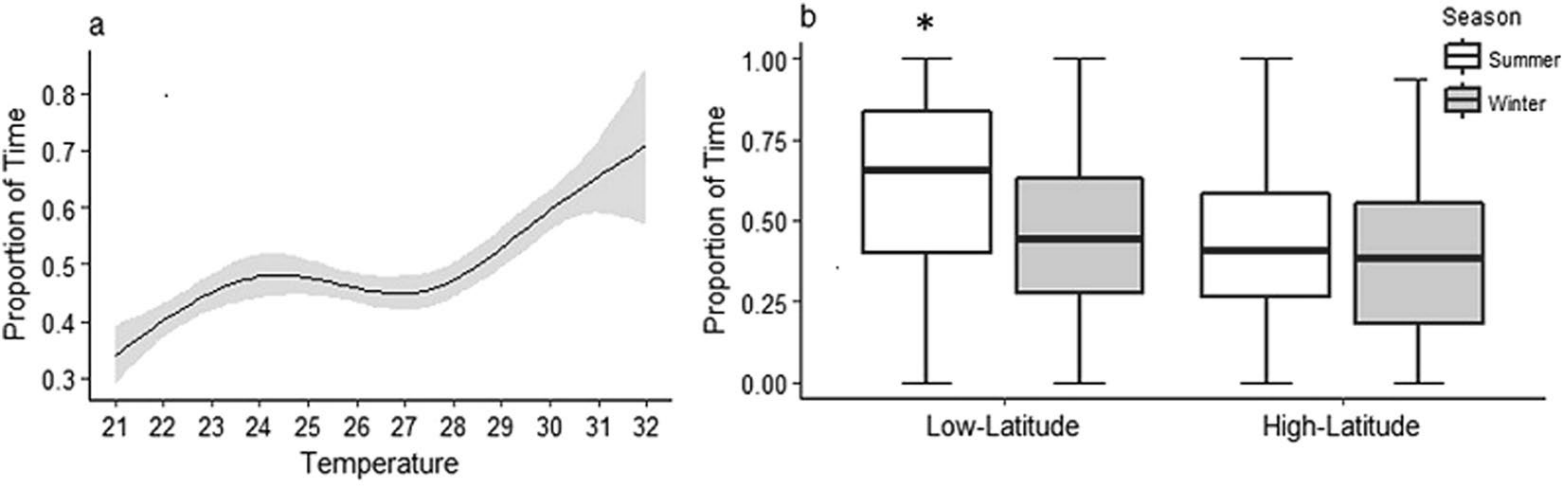
(**a**) Modelled values of a Generalised Additive Model with a smoothing function displaying the relationship between the proportion of time *P. leopardus* spent stationary with increasing temperature (p < 0.01). The shaded region around the curve represents a 95% confidence interval. (**b**) Box plots demonstrating the median proportion of time low-latitude and high-latitude populations of *P. leopardus* spent stationary (p < 0.01) during summer (white) and winter (grey) **(**p < 0.01). Data (n = 595) are for each individual observation. The whiskers are extended to extreme values. Linear models were used to test for differences in time spent stationary between location and season. Significant differences are marked with an *.

The proportion of time an individual spent stationary increased with increasing temperature (Fig. 4a, Table 2). On average, the time spent resting increased from 25.3 ± 0.03% at 21 °C to 90.6 ± 0.05% at 32 °C. This behaviour was most pronounced for the low-latitude population in the summer, who spent approximately 62% of their time inactive, compared with 47% in the winter. In contrast, the high-latitude population in summer spent 43% of their time inactive compared with 37% in winter (Fig. 4b).

**Figure 5:**
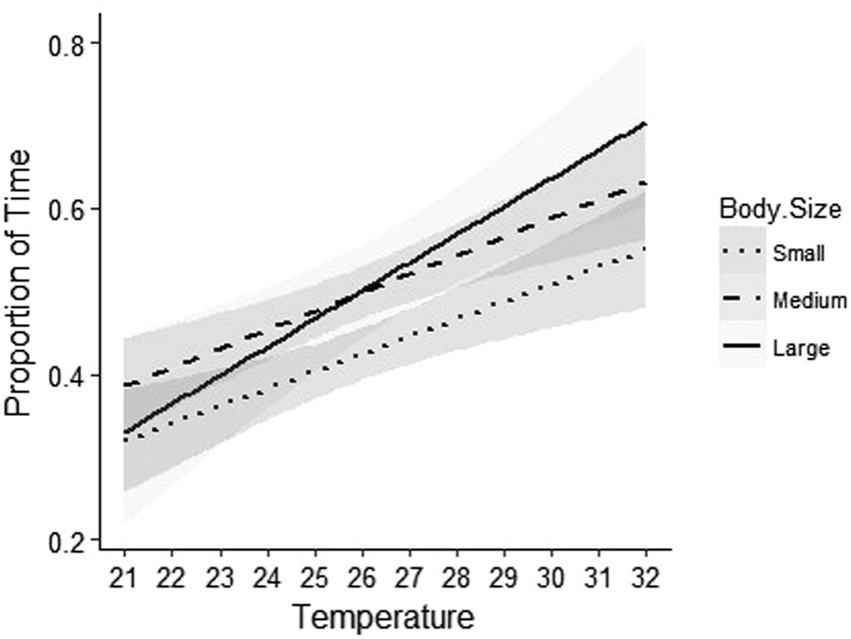
Plot of the modelled values from a linear regression showing the interaction between the proportion of time spent resting at increasing temperatures with body size (measured as total length to the nearest 5 cm) of *P. leopardus* with small (10–30 cm, TL, dotted line), medium sized (35–45 cm, TL, dashed line) large body size (>50 cm, TL, bold line), (p < 0.001).

The proportion of time spent inactive was further influenced by body size (p < 0.001) (Table 2), with medium (35–45 cm, TL) and larger (>50 cm, TL) individuals spending a greater proportion of time inactive than smaller individuals (<35 cm, TL). The impact of water temperature on activity patterns was greatest for larger individuals (Fig. 5).

## Discussion

The effects of global warming on large predatory and commercially important coral reef fishes is critically important given the potential of increasing ocean temperatures to compromise fitness and performance of coral reef fisheries species^31^. Given that fishes are ectotherms, increases in ocean temperature will lead to inevitable increases in baseline metabolic rates^6^ which may be partially compensated for through increased food intake. In this study, we show that strike rates by *P. leopardus* increased from 0.015 strikes per hour at 21 °C up to 0.023 strikes per hour at 30 °C equating to a 1.4 – fold increase in strike rate for every 3 °C temperature rise. This increase is consistent with the expected 1.2–1.4 fold increase in energy need associated with a 3 °C temperature rise identified in previous studies^11,40^. However, strike rates did not increase beyond 30 °C, suggesting that *P. leopardus* may not be able to compensate for temperature induced increases in metabolic rate beyond this threshold, which closely corresponds with the mean maximum temperature to which fishes are already exposed from low-latitude regions on the GBR^25^. Constraints on food intake with projected increases in ambient temperatures from low-latitude regions^1^ may be further compounded by limited food availability as well as constraints on energetic expenditure and movement.

Increased food intake by *P. leopardus* will almost certainly require increased foraging activity and energy expenditure. Conversely, temperature-induced increases in basic metabolic demands will reduce energy available for movement and feeding. Our data show that the proportion of time that *P. leopardus* are inactive increases with increasing temperature from 21 to 32 °C. Already, fish from low-latitude regions of the GBR spend a significant proportion of their time completely inactive when exposed to high temperatures during summer. These behavioural changes have potentially widespread implications, not only for the fitness of individuals but also for population dynamics and ecosystem function under warming oceans^41^. Any reductions in swimming and activity patterns are likely to not only influence foraging efficiency and the ability to capture prey^42,43^, but also increase the risk of predation, and potentially influence species demography through changes to longer term activity patterns and space use^44,45^. Importantly, *P. leopardus* are known to undertake periodic spawning related movements^46^. Decreased mobility and a greater need to conserve energy may potentially reduce overall space use and reproduction^47^, which could directly influence population replenishment and the viability of fisheries stocks, especially given larger-bodied individuals are likely to be disproportionately impacted^39^ if they are unable to seek thermal refuge.

In this study, larger individuals (>50 cm, TL) exhibited a more pronounced response to increasing temperatures and spent proportionally more time inactive than their smaller conspecifics. Larger individuals are considered to be more thermally sensitive than smaller individuals due to size-dependent oxygen limitation to tissues and organs meaning that temperature-dependent aerobic limits are experienced earlier by larger individuals^48,49^. This pattern has been demonstrated for *P. leopardus* under laboratory conditions^39^, and is consistent with slower swimming speeds and longer resting times found in large *P. leopardus* at elevated temperature^10^. Given the predicted vulnerability of large-bodied species to temperature rise, recent studies have suggested a warming-induced trend towards smaller adult size classes as a response to global warming^49,50^. A reduction in predator size, may necessitate selection for smaller prey items, which may impact the size distributions of smaller reef fishes, potentially altering food webs and population dynamics^51^. In this study, smaller individuals had consistently higher strike rates than larger individuals, and this pattern was unaffected by temperature. Johansen *et al*. (2015) demonstrated a similar response, that relative to body size, small and medium sized *P. leopardus* consumed more food than larger individuals^11^. Smaller individuals typically have higher mass-specific metabolic rates than larger individuals, which may be associated with higher growth rates and elevated activity levels^52,53^. However, increased foraging efficiency of smaller individuals may come at a cost, as energy expenditure and risk of predation may increase with foraging frequency^54^.

The differential effects of temperature on body size may modify predator-prey interactions by impacting predation success or prey escape response^20,55^. If increasing temperatures have a disproportionate impact on larger bodied individuals or species^39,52^, the capacity of predators to exert the necessary energy may be increasingly constrained while prey may be better able to escape predators^20^. Alternatively, prey may exhibit a decreased escape response at elevated temperature, increasing capture success by predators^55^. Differences in the temperature dependence of predator-prey interactions may lead to changes in trophodynamics, community structure and function.

Whilst individual plasticity in foraging behaviour is likely to compensate for increased metabolic demands in *P. leopardus* exposed to moderate increases in temperature, it appears that individuals may be adversely affected by temperatures >30 °C^56^. Notably, *P. leopardus* from low-latitude regions of the GBR are already exposed to summer temperatures >30 °C^25^. Even slight declines in strike rates and foraging efficiency at higher temperatures, compounded by a substantial reduction in movement and activity patterns, suggest that *P. leopardus* may have limited capacity to cope with projected increases in temperatures due to climate change. Low-latitude populations of *P. leopardus* are therefore expected to be particularly vulnerable to increases in ocean temperature. Unless fish are able to seek thermally favourable habitats by moving to cooler, deeper waters, or shift their distribution to higher latitudes, physiological limits^31,39^ and food availability^11^ may constrain their capacity to endure longer-term and more severe ocean warming^57^.

This study, is the first of its kind to demonstrate a predatory coral reef fish species modifies its foraging behaviour and activity *in situ* in response to seasonal and latitudinal differences in temperature. The combination of our data and previous laboratory studies of *P. leopardus* ^10,11,31,39^ provide a holistic overview of the temperature dependence of behavioural and physiological performance of a coral reef predator. *P. leopardus* play a significant role in structuring fish communities and maintaining ecosystem health^58,59^. Any alterations to their feeding patterns and activity may therefore have significant implications for trophic food webs and consequently ecosystem function. If *P. leopardus* are unable to adapt, acclimate, or acclimatise to increasing temperatures (behaviourally or physiologically) it is likely that the fitness of *P. leopardus* populations on the GBR, especially in the low-latitude region, may be undermined by continued increases in ocean temperature. Further research is needed to investigate how these individual level effects scale up to affect whole communities and over spatial and temporal time scales relevant to the pace of climate change.

## Methods

This work was approved by the Animal Ethics Committee (AEC) James Cook University and carried out in accordance with James Cook Animal Ethics Approval No. A2310.

### Study location

**Figure 6:**
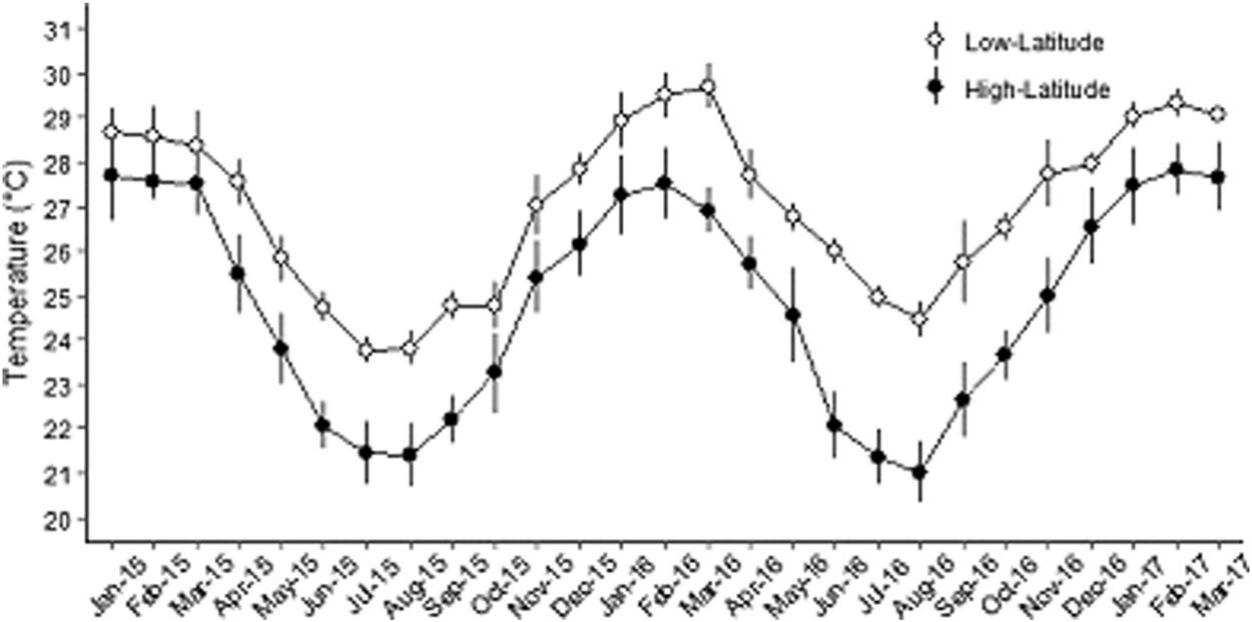
The minimum and maximum water temperatures from the low-latitude location (white) and high-latitude location (black) from January 2015–March 2017.

This study was conducted across two latitudinally distinct locations on Australia’s Great Barrier Reef (GBR); Lizard Island (14°40′S, 145°27′E) in the northern GBR (low-latitude population) and Heron Island (23°29′S, 151°52E) in the southern GBR (high-latitude population). The locations are separated by approximately 1,200 km and 10 degrees of latitude. Sampling was conducted in summer (February–March 2016) and winter (July–August 2016) to encompass maximum and minimum annual temperatures experienced by each population of *P. leopardus* (Fig. 6). Each location was situated within a ‘Marine Park’ zone on the GBR implying a negligible impact of fishing pressure at both locations. Specific sampling was conducted within comparable coral reef habitat at each location, and all surveys were conducted along the shallow reef crest and adjacent slope areas < 10 m. Temperature was recorded from dive computers which are accurate to 0.01 degrees.

### Foraging behaviour

A strike was determined if a *P. leopardus* was observed making an uncharacteristically fast, i.e. >1 body length per second, purposeful burst towards a prey item^55^. To test for diurnal variation in feeding behaviour of *P. leopardus*, field observations were undertaken within three distinct time periods: morning (0700–1100 hrs, n = 290), midday (1101–1400 hrs, n = 187) and afternoon (1401–1730 hrs, n = 118). Sites at each location were chosen haphazardly and 2–3 sites were sampled each day. At each site, 3–5 trout observations were made by 2 observers giving an average of 10–12 trout observations per day. For each sampling period between 125 and 164 individual fish observations were made, giving a total of 595 observations. To reduce observer bias, each observer was given a 60 minute guided observation by the chief investigator to ensure all observers were observing and recording *P. leopardus* behaviour accurately. Upon entering the water, the first *P. leopardus* found was chosen and observed for up to 60 minutes at a distance >5 m. These parameters were chosen based on previous observational studies of coral trout (pers. comm. A. Vail). This distance caused no apparent distress to the fish, and fish appeared to behave normally (as per Sweatman 1984^60^). An individual trout was followed on snorkel or SCUBA at a random depth between 1–10 m and the number of strikes were recorded. Where possible, observations were conducted for 60 minutes, but even where fish were lost or observations aborted, data was retained as long as the observation period was >15 minutes. This allowed for strike rate (number of strikes per unit of time observed) to be measured as a proxy for foraging behaviour. Other variables measured were: water temperature (°C), total length of the individual (to the nearest 5 cm), type of habitat over which the individual was hunting, the distance over which the individual moved to hunt prey (m), depth of the hunt (m), visibility (m), and the outcome of the predation event. Predation success was recorded by all observers. However, to reduce observer bias, only the primary observer’s data were used in statistical analysis. In addition to foraging behaviour, the amount of time an individual spent stationary or inactive throughout the observation was recorded, enabling a measurement of the proportion of time spent resting.

### Data availability

The datasets generated and/or analysed during the study are available from the corresponding author on reasonable request.

### Statistical analysis

Spatial and temporal variation in strike rates of *P. leopardus* were examined using a negative binomial generalized linear model from the package ‘*MASS’* in R Statistical Software™. Variance inflation factors (VIF) were calculated to determine the multicollinearity of the variables location, temperature and season. Season and temperature had VIF >5 so season was included in all models, and the data were centered around temperature to reduce collinearity. Other predictors tested were; body size, location, method of observation (i.e. snorkel or scuba), and time of day on strike rates. Negative binomial regression is useful for modelling count variables, with a moderate proportion of zeros, particularly if they are overdispersed^61,62^. Coefficients from the negative binomial correlation of coefficients table (z-values) were converted to p-values. A generalized additive model (GAM) was then used to separately analyse the relationship between temperature and body size as continuous predictors against strike rate, which was expected to be non-linear. GAM’s allow for non-linear relationships between the response variable and explanatory variables and for the combination of both linear and complex additive responses by adding a smoothing curve through the data. The *‘mgcv’* package was used because it allows for cross-validation, a process that automatically determines the optimal amount of smoothing. To determine the differences in success rates between seasons, locations and size class a generalized linear model with quasibinomial distribution (chosen when the response variable is a proportion) and a logit link function was used and the best fit model was selected according to Akaike Information Criteria (AIC). Differences in strike rate with habitat were analysed by a one-way ANOVA comparing strike rates between 3 habitat groups; reef matrix, water column, and other. To analyse *P. leopardus* resting behaviour, a GAM tested the proportion of time spent resting in relation to temperature and body size which were treated as continuous variables. All analyses were performed in the R-Environment^62^.

## Electronic supplementary material

Supplementary Material
