# Supplementary material for: A large predatory reef fish species moderates feeding and activity patterns in response to seasonal and latitudinal temperature variation
Source: Sci Rep. 2017 Oct 11;7:12966. doi: 10.1038/s41598-017-13277-4 (PMC5636919; doi:10.1038/s41598-017-13277-4)
Supplement: Supplementary file 1 — Supplementary Material [file 41598_2017_13277_MOESM1_ESM.pdf]

# A large predatory reef fish species moderates feeding and activity patterns in response to seasonal and latitudinal temperature variation

Authors:* Molly Scott[1], Michelle Heupel[2], Andrew Tobin[3], Morgan Pratchett[1]

1 ARC Centre of Excellence for Coral Reef Studies, James Cook University, Townsville QLD 4811, Australia.
2 Australian Institute of Marine Science, PMB No 3, Townsville, Queensland 4810, Australia
3 Centre for Sustainable Tropical Fisheries and Aquaculture and School of Earth and Environmental Sciences, James Cook University, Townsville, Queensland 4811, Australia
*Corresponding author Molly Scott. (email: molly.scott@my.jcu.edu.au)

## Supplementary Material

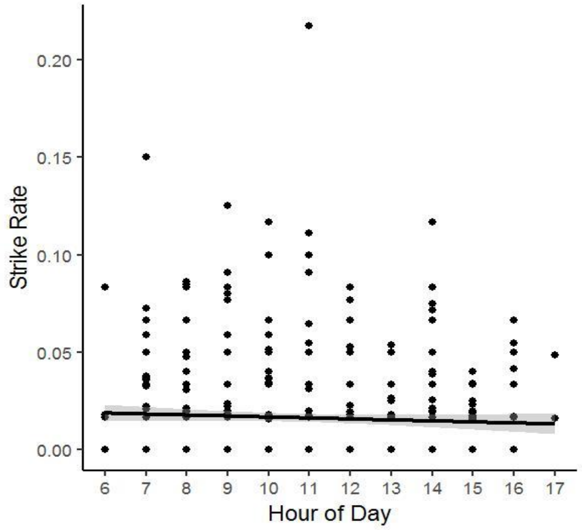


Figure S1: The modelled values of a generalised linear model illustrating that strike rates of *P. leopardus* were not significantly different between hours of the day (p = 0.796).

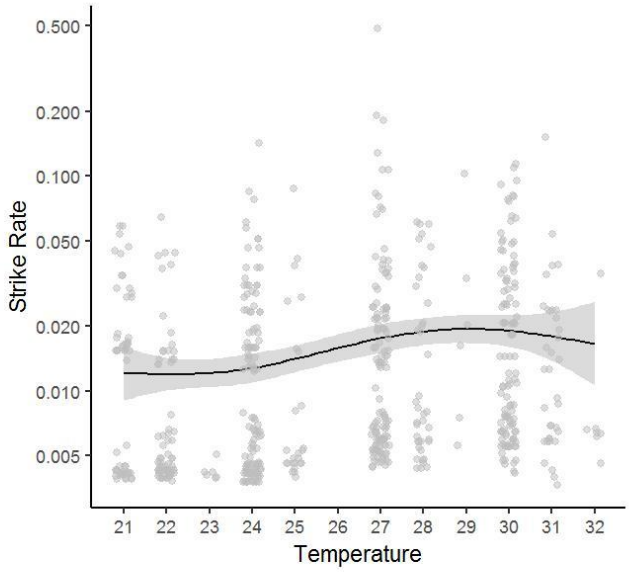


Figure S2: The modelled values of the relationship between strike rate and increasing temperature (p < 0.01) from a Generalised Additive Model with a smoothing function. The shaded region around the curve represents a 95% confidence interval, the grey points are residual values from the fitted model and have been jittered to show their variation.
